# A validated stability-indicating HPLC method for determination of brimonidine tartrate in BRI/PHEMA drug delivery systems

**DOI:** 10.1186/s13065-017-0292-2

**Published:** 2017-07-11

**Authors:** Jianguo Sun, Xiuwen Zhang, Taomin Huang

**Affiliations:** 1 Eye Institute, Eye & ENT Hospital, Shanghai Medical College, Fudan University, Shanghai, 200031 China; 2 Department of Pharmacy, Eye & ENT Hospital, Shanghai Medical College, Fudan University, Shanghai, 200031 China; 3 Key Laboratory of Myopia, NHFPC, and Shanghai Key Laboratory of Visual Impairment and Restoration, Fudan University, Shanghai, 200031 China

**Keywords:** Liquid chromatography, Method validation, Brimonidine tartrate, Impurities, Drug delivery system, Contact lens

## Abstract

**Background:**

A simple, rapid and accurate stability-indicating reverse phase high performance liquid chromatography (RP-HPLC) was developed and validated for the determination of brimonidine tartrate in brimonidine tartrate/poly(2-hydroxyethyl methacrylate) (BRI/PHEMA) drug delivery contact lenses and pharmaceutical formulations.

**Results:**

Optimum chromatographic conditions for separating brimonidine tartrate from other impurities in the leaching liquor of BRI/PHEMA drug delivery contact lenses or pharmaceutical formulations have been achieved by using a Diamonsil C18 column (150 mm × 4.6 mm, 5 μm) as a stationary phase and a mixture solution of phosphate buffer (10 mM, pH3.5) containing 0.5% triethlamine and methanol (85:15, v/v) as a mobile phase at a flow rate of 1 mL/min. The theoretical plates for the brimonidine tartrate measurement were calculated to be 8360 when detection was performed at 246 nm using a diode array detector. The proposed method was validated in accordance with ICH guidelines with respect to linearity, accuracy, precision, robustness, specificity, limit of detection and quantitation. Regression analysis showed a good correlation (R^2^ > 0.999) for brimonidine tartrate in the concentration range of 0.01–50 μg/mL. The peak purity factor is ≥980 for the analyte after all types of stress tests, indicating an excellent separation of brimonidine tartrate peak from other impurities. The measurement course could be completed within 10 min, which was very quick, effective and convenient.

**Conclusions:**

Overall, the proposed stability-indicating method was suitable for routine quality control and drug analysis of brimonidine tartrate in BRI/PHEMA drug delivery contact lenses and other pharmaceutical formulations.

## Introduction

Glaucoma is an ocular disease characterized by elevated intraocular pressure (IOP) and progressive optic neuropathy, leading to visual loss [1]. Decreasing and maintaining IOP by means of topical drug administration is the most direct and preferred treatment options to treat glaucoma. Brimonidine [5-bromo-6-(2-imidazolidinylideneamino) quinoxaline] is a highly selective α_2_-adrenoceptor agonist which can lower IOP and is approved for topical ocular administration for glaucoma treatments.

**Fig. 1:**
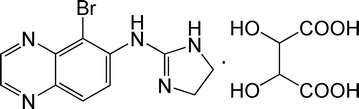
The structural formulae of brimonidine tartrate

Brimonidine tartrate (shown in Fig. 1) can not only lower IOP [2], but also protective optic nerve and thus limit the progression of visual loss in glaucoma [3]. However, the topical administration of brimonidine tartrate eye drops has low bioavailability through the cornea (1–7%), and the remaining drug which enters systemic circulation can cause side effects [4]. Moreover, the application of ophthalmic drugs as drops results in a fluctuating concentration of drug penetrated into the cornea, and thus limits their therapeutic efficacy. It is difficult to achieve sustained therapeutic level of topically applied ophthalmic drugs in the eye because of structural and metabolic barriers, especially in the vitreous and retina. Therefore, new types of drug delivery systems are highly desirable to increase drug delivery efficacy and reduce side effects, and to improve the drug therapeutic effect by controlling the rate of drug delivery.

Hydrogel-based contact lenses were used to prepare local drug delivery systems for treating glaucoma which consisted of a swellable polymer hydrogel and commonly used glaucoma drugs [5–7]. Poly(2-hydroxyethyl methacrylate) (PHEMA) hydrogels are well known as materials for contact lenses and ophthalmic implanted materials [8, 9], and their potential uses as drug delivery carriers have also been reported in recent research [8, 10]. Ophthalmic drug delivery via PHEMA contact lenses could improve the delivery efficiency by increasing the residence time of drug on the eye surface and simultaneously reducing drug wastage and side effects [11]. Brimonidine tartrate can be delivered to the post-lens tear film by wearing a PHEMA contact lens which was prepared by molecular imprinting polymerizing technique [12], or only by simple soaking absorption and release method [13]. Drug could be sustainably released from the PHEMA contact lens for several hours or days. Thus, the drug should be stable in the contact lens. In the in vitro drug release course, apart from brimonidine tartrate, some impurities, such as unreacted 2-hydroxyethyl methacrylate, cross linker or polymerization initiator, might be released into the leaching liquor, which might affect the measurement of the purpose brimonidine tartrate. Therefore, the stability study of brimonidine tartrate is urgent and it is necessary to develop a rapid and efficient method to quantitatively analyze brimonidine tartrate released from BRI/PHEMA contact lenses.

Various analytical methods have been reported for the determination of brimonidine tartrate, including electroanalytical method [14, 15], spectrophotometric method [16], highly sensitive gas chromatography/mass spectrometric assay [17], high-performance thin layer chromatography (HPTLC) [18], high-performance liquid chromatography (HPLC) [19–21], liquid chromatography–mass spectrometry (LC–MS) [22, 23] and high performance liquid chromatography-tandem mass spectrometry (HPLC-TMS) [24]. However, some of the above-described methods are limited in either low sensitivity or specificity. Furthermore, extensive survey revealed that no stability-indicating high performance liquid chromatography (HPLC) method has been reported including major pharmacopoeias such as USP, EP, JP and BP for the simultaneous determination of brimonidine tartrate and other impurities. Therefore, it is very promising and urgent to develop a stability-indicating HPLC method to simultaneously determine brimonidine tartrate and its impurities in the leaching liquor of BRI/PHEMA contact lenses. So we first prepared BRI/PHEMA contact lenses by photopolymerization of 2-hydroxyethyl methacrylate, brimonidine tartrate and cross linker assisted by polymerization initiator. Some leaching liquor of BRI/PHEMA contact lenses in the drug release course was collected and then a rapid and efficient RP-HPLC method was developed for the determination of brimonidine tartrate and other impurities in the leaching liquor of BRI/PHEMA contact lenses. Linearity, accuracy, precision, specificity, robustness, LOD and LOQ of the proposed method were demonstrated based on method validation.

## Methods

### Materials and reagents

This process used 2-hydroxyethyl methacrylate (HEMA) (J & K Chemical Ltd. Shanghai, China). Poly (ethylene glycol) dimethacrylate (PEG-DMA, MW700), brimonidine tartrate (Lot No.: LA50Q41, BRI) and 2-hydroxy-1-[4-(hydroxyethoxy) phenyl]-2-methl-1-propanone (D2959) were purchased from Sigma-Aldrich (Shanghai, China). Brimonidine tartrate eye drops (Alphagan, 0.2% brimonidine tartrate, w/w) were obtained from Allergan Pharmaceuticals (Republic of Ireland). Potassium dihydrogen phosphate (KH_2_PO_4_) was obtained from Sinopharm Chemical Reagent Co. Ltd. (Shanghai, China). Phosphoric acid was obtained from Lingfeng Chemical Reagent Co. Ltd. (Shanghai, China). Triethlamine (HPLC grade) was obtained from Fisher scientific (New Jersey, USA). HPLC-grade methanol was obtained from TEDIA (OH, USA). All above chemicals were analytical grade and used as received. All solutions were prepared in Milli-Q deionized water from a Millipore water purification system (Bedford, MA, USA). Mobile phase was filtered using 0.45 µm nylon filters from Millipore Co. (MA, USA) by an Auto Science AP-01P system from Tianjin Automatic Science Instrument Co. LTD (China).

### Preparation of sample solutions

The BRI/PHEMA contact lens was prepared by a UV light polymerization reaction as reported previously [9, 25]. Briefly, 6 g of 2-hydroxyethyl methacrylate (HEMA) monomer solution was mixed with 90 mg of brimonidine tartrate, 180 mg of PEG-DMA as a cross linker and 18 mg of D2959 in a 15-mL brown glass bottle and the mixture was gently stirred under N_2_ gas for 20 min. A polydimethylsiloxane (PDMS) mold which has a spherical cavity was used to prepare a PHEMA contact lens. The mixture solution was injected into the PDMS mold and the upper mold was slowly covered onto the lower mold to remove air bubble. Then the mold was placed vertically in UV light (365 nm, SB-100P/F, Spectronics Corporation, USA) for 30 min to polymerize a BRI/PHEMA composite film. The film was peeled from the mold and then carefully tailored into a contact lens. As comparison, the pure PHEMA contact lens was prepared similarly by the same method.

ABRI/PHEMA contact lens sample (~0.2 g) was placed into a 50-mL plastic container with 30 mL of phosphate buffered saline (PBS) solution (pH = 7.4). The container was firmly capped with the lid and shaken at 37 °C and a speed of 50 rpm in the DKZ-3B shaker (Shanghai Yiheng Scientific instruments Co. Ltd.). At 1 h, 24 h and 7 day, 1 mL of the leaching liquor of the BRI/PHEMA contact lens was collected and the same volume was supplied. The pH of the leaching liquor was adjusted to 3.5 with 1 M HCl and filtered with a 0.45 μm nylon filter and centrifuged twice to remove any undissolved substance before the quantitative measurement by HPLC. Similarly, the leaching liquor of a pure PHEMA contact lens was also collected as a blank control.

### Preparation of standard solution

A standard stock solution of brimonidine tartrate (1 mg/mL) was prepared using the mobile phase. Series working solutions were diluted to the desired concentration for linearity, accuracy, precision, solution stability and robustness etc.

### Equipment and chromatographic conditions

Samples were analyzed on an Agilent 1100 HPLC system (Agilent Technologies, Palo Alto, CA, USA), attached with a G1311A quaternary pump, a G1312A vacuum degasser, and a G1315B DAD detector. The detector wavelength was fixed at 246 nm and the peak areas were integrated automatically using the Hewlett–Packard ChemStation software program [16]. Other apparatus included an ultrasound generator and a SevenEasy pH meter (Mettler Toledo, USA) that was equipped with a combined glass–calomel electrode. A Diamonsil C18 column (150 mm × 4.6 mm, 5 μm) was maintained at 30 °C. The mobile phase was composed of a phosphate buffer (10 mM, pH 3.5) containing 0.5% triethlamine and methanol (85:15, v/v). The flow rate of the mobile phase was set at 1 mL/min. Measurements were made with 20 μL of injection volume. For the analysis of the forced degradation samples, the photodiode array detector was used in a scan mode with a range of 200–400 nm. The peak homogeneity was expressed in terms of peak purity factor and was obtained directly from the spectral analysis report using the above-mentioned software.

### Method validation

The proposed method was validated according to ICH guidelines [26] including linearity, accuracy, precision, specificity, robustness, limit of detection (LOD) and limit of quantitation (LOQ). The linearity test solution was freshly prepared by diluting the stock standard solution with mobile phase. The linearity was tested at six levels ranging in 0.01–50 μg/mL (0.01, 0.1, 0.5, 1, 10, 25, 50 μg/mL) for brimonidine tartrate. Each solution was prepared in triplicate. Calibration curves were plotted between the responses of peak versus analyte concentrations. The coefficient correlation, slope and intercept of the calibration curve were calculated. Accuracy of the developed method was determined by standard addition method. For this purpose, known quantities of brimonidine tartrate (0.1, 10, 50 μg/mL) were supplemented to the sample solution previously analyzed. Then, the experimental and true values were compared. The precision was tested by intra-day and inter-day precision at three level concentrations (0.1, 10, 50 μg/mL). Intra-day precision was studied on the same day (*n* = 5). And inter-day precision was determined by performing the same procedures on three consecutive days. Percentage relative standard deviation (RSD %) for peak areas was then calculated to represent precision. Specificity was the ability of the method to measure the analyte from the excipients and potential impurities. The specificity of the developed method was investigated in the presence of potential impurities. To determine the robustness of the developed method, the mobile phase composition, flow rate and pH value of buffer solution were deliberately changed. The effects of these changes on chromatographic parameters such as retention time, symmetry and number of theoretical plates were then investigated. LOD and LOQ values were determined at signal-to-noise (S/N) ratios of 3:1 and 10:1, respectively, by measuring a series of dilute solutions with known concentrations.

### Forced degradation studies

Forced degradation studies were carried out using differently prescribed stress conditions such as thermolytic, photolytic, acid, base hydrolytic and oxidative stress conditions according to a previously reported method [27–29].

#### Acid degradation

For this purpose, 2.5 mL of the standard stock solution was transferred into a 100 mL volumetric flask. And then 2.5 mL of 5 M HCl was added into the flask, which was kept at 40 °C for 24 h in water bath. After completion of the acid stress, the solution was cooled in room temperature and neutralized by 5 M NaOH and the volume was completed up to the mark (100 mL) with mobile phase.

#### Alkali degradation

In a 100-mL volumetric flask, 2.5 mL of the standard stock solution was added. Then 2.5 mL of 5 M NaOH was also added into the flask and the solution was kept at 40 °C for 2 h in water bath. The solution was cooled in room temperature and neutralized by using 5 M HCl and diluted to the mark (100 mL) with mobile phase.

#### Oxidative degradation

For this purpose, 2.5 mL of the standard stock solution was transferred into 100-mL volumetric flask. 2.5 mL of 6% H_2_O_2_ was added into the flask and keep at 40 °C for 24 h in water bath. Then, the solution was cooled in room temperature and diluted to the mark (100 mL) with mobile phase.

#### Thermal degradation

Thermal degradation study was performed at two different temperatures: 40 °C in an electric-heated thermostatic water bath (DK-S28) and 105 °C in oven (dry heat thermolysis, DGH-9203A), which were both from Shanghai Jing Hong Laboratory Instrument Co. Ltd. (China). For thermal degradation at 40 °C, 2.5 mL of the standard stock solution was transferred into 100-mL volumetric flask and kept at 40 °C in water bath for 120 and 240 h. The solution was cooled in room temperature and the volume was completed up to the mark with mobile phase. Dry heat thermolysis was conducted by taking standard brimonidine tartrate in Petri dish and heated in oven at 105 °C for 7 h. After completion of the stress, the powder was dissolved and diluted with mobile phase. Photo stability studies were performed on a photo stability test chamber.

#### Photolytic degradation

Photolytic degradation study was conducted by exposing samples in a photo-stability test chamber (Pharma 500-L, Weiss Technik UK Ltd. Germany) at 1.2 million lux hour for light and 200 Wh/m^2^ for ultraviolet region. After photolytic degradation, samples were diluted with mobile phase to achieve a concentration of 25 μg/mL and injected into the HPLC measurement system.

## Results and discussion

### Preparation of BRI/PHEMA contact lens

**Fig. 2:**
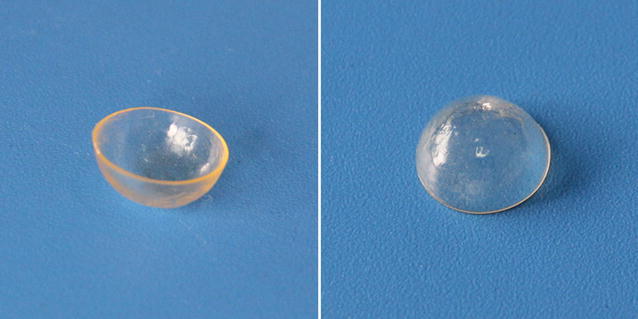
Photos of hard contact lens

In the preparation course of a BRI/PHEMA contact lens, the monomer HEMA and the PEG-DMA were co-polymerized by initiating with a UV light free radical initiator D2959. Using this initiator system, a PHEMA film can be obtained at room temperature. The BRI/PHEMA film prepared using UV-light copolymerization was visually transparent, indicating brimonidine tartrate was well dispersed in the composite film. The resultant PHEMA and BRI/PHEMA films could be tailored into hard contact lenses which are shown in Fig. 2. The hard contact lens could further form soft-hydrogel contact lens after swelled in water.

A range of UV light radiation time (10–40 min) was tested for the HEMA polymerization, and time of 30 min was found to be sufficient to obtain a BRI/PHEMA film with smooth surface. When the BRI/PHEMA contact lens sample was leached in PBS solution (pH = 7.4, 37 °C) for 1 week, the purpose sample solution was collected for the quantitative analysis of brimonidine tartrate loaded in the BRI/PHEMA contact lenses.

### Optimization of the chromatographic system

**Fig. 3:**
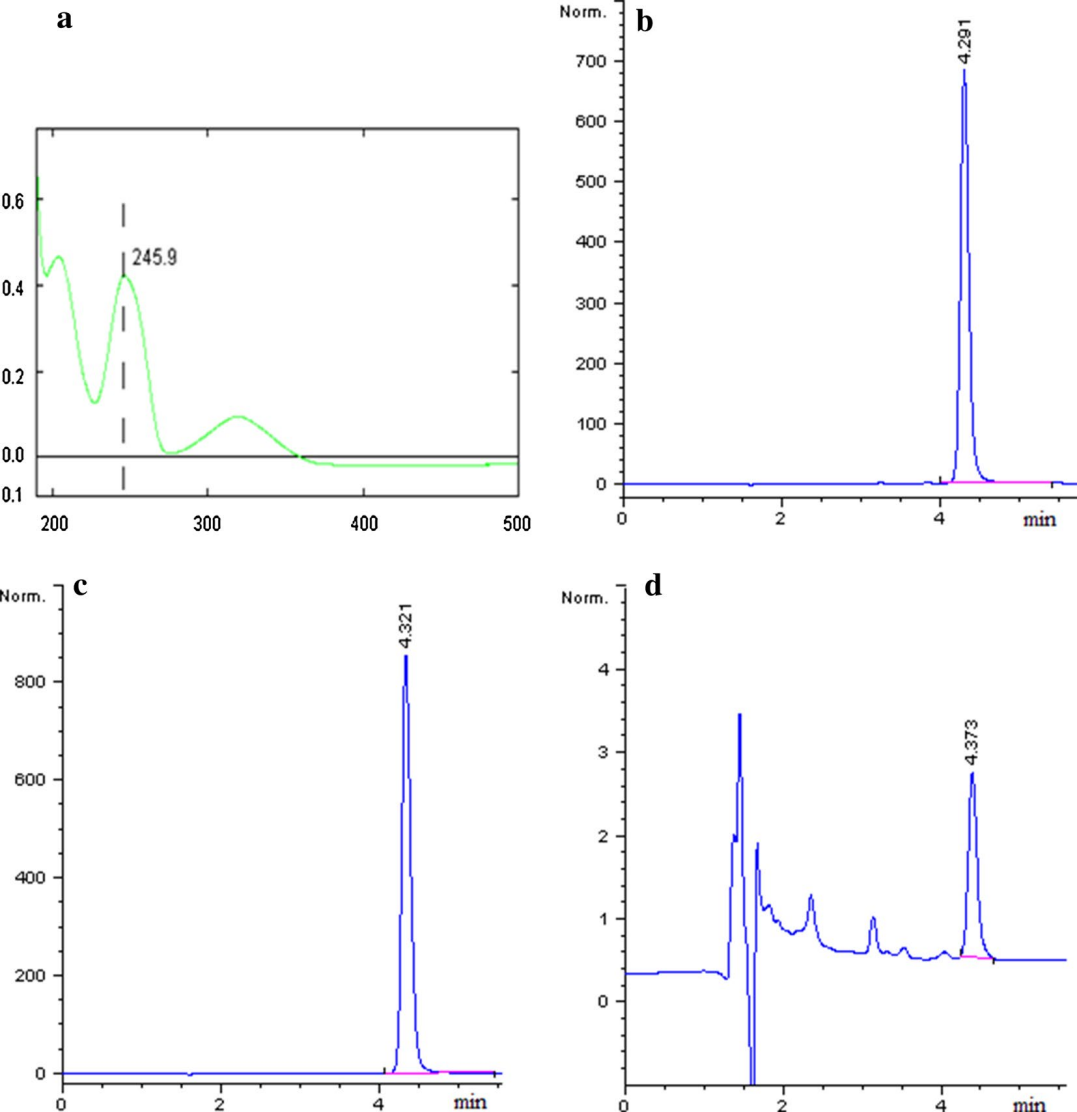
The UV spectrum and chromatograms of brimonidine tartrate. **a** UV spectrum of brimonidine tartrate; **b** chromatogram for separation of brimonidine tartrate in standard solution; **c** chromatogram of brimonidine tartrate in commercial ophthalmic solution; **d** chromatogram of brimonidine tartrate in BRI/PHEMA formulation

The main objective of this work was to develop a stability-indicating HPLC method for determination of brimonidine tartrate within a short run time between 3–10 min and symmetry between 0.80 and 1.20. The pKa of brimonidine is 7.4, it will be substantially ionized at pH below 6.5. Therefore, brimonidine tartrate can be ionized as brimonidine positive ion in the mobile phase (pH 3.5). UV spectrum (Fig. 3a) showed that brimonidine tartrate has a characteristic absorption peak at 245.9 (~246) nm. The chromatographic peak of brimonidine positive ion was about 4.3 min which was shown in Fig. 3b. Chromatograms of brimonidine tartrate in commercial ophthalmic solution and BRI/PHEMA formulation were shown in Fig. 3c, d, respectively. The content of brimonidine tartrate was calculated according to the peak area of brimonidine tartrate (about 4.3 min) in this study.

Brimonidine tartrate has high ratio of carbon to heteroatom and has conjugated bond. Therefore, they can be separated through C18 stationary phase mainly based on their overall hydrophobicity. Brimonidine tartrate can also be separated using phenyl-hexyl stationary phase considering their π electrons involving π–π interactions. So they may be separated using cyano stationary phase.

The stationary and mobile phases play an important role on theoretical plates, peak shape, symmetry and resolution. To obtain symmetrical peaks with better resolution and no peak impurity, various chromatographic conditions were investigated and optimized for the determination of brimonidine tartrate, such as mobile phases with different composition, pH and stationary phases with different packing material etc. Attempts were made by using three kinds of HPLC columns (Agilent Zorbax Eclipse XDB C18, Agilent Eclipse Plus Phenyl-Hexyl and Diamonsil C18 column) with different mobile phase compositions and ratios. In all of the proceeding columns, broad characteristic peaks were obtained though using different ratios (20:80, 40:60, 50:50, 70:30, 80:20) of methanol/acetonitrile and water. No improvement of peak shapes was obtained even when the temperature of column was enhanced to 40 °C. Some data of composition optimization of mobile phases were shown in Table 1, in which a Diamonsil C18 column was used.

**Table 1:** The optimisation of the mobil phases of solvent ratios with the Diamonsil C18 column

Mobile phase	Theoretical plates (N)	Symmetry	Peak shape
Methanol:water = 15:85	476	0.78	–
Acetonitrile:water = 15:85	607	0.70	–
0.010 M KH_2_PO_4_:water = 15:85	6483	0.63	–
0.025 M KH_2_PO_4_:water = 15:85	6258	0.61	–
0.050 M KH_2_PO_4_:water = 15:85	6616	0.66	–

–, poor peak shape

As demonstrated in Table 1, the theoretical plates with the mixture solution of methanol or acetonitrile with water as a mobile phase were below 1000 which indicated poor column chromatography separation power. The peak symmetry and peak shape were all poor with the above two kinds of mixture solutions, which might be attributed to low polarity of the mobile phase. So some phosphate buffer with different concentration (10, 25 or 50 mM) was used to improve polarity of the mobile phase, which resulted in a narrowed peak. However, the peak shape and peak symmetry were still not satisfactory. So some triethylamine (as silanol blocker) was further added to the above polar mobile phase to improve the separation of brimonidine tartrate with other impurities. Finally, the mixture solution of phosphate buffer (10 mM), trimethylamine (0.5%, v/v) and methanol (15%, v/v) was demonstrated to be the suitable mobile phase for the improvement of peak shape and peak symmetry. With exception of the composition of mixture solution, buffer pH was also found to be critical in the analyte separation and method optimization. The effect of buffer pH on retention time was related with the ionization form of the solute. A series of mixture solutions with different pH values (2.5, 2.8, 3.0, 3.5, 4.0, 5.0, 6.0, 7.0 and 8.0) were employed to investigate the retention time and resolution of brimonidine tartrate with other impurities in pharmaceutical formulation, in which the other chromatographic parameters were kept unchanged, including a Diamonsil C18 column and the fixed mobile phase composition of phosphate buffer (10 mM), trimethylamine (0.5%, v/v) and methanol (15%, v/v).

As shown in Table 2, a buffer solution with pH of 3.5 was found to be optimal with more theoretical plates (≥8360), narrow peak (+++), high peak symmetry (0.95) and short retention time (4.3, between 3 and 10 min), which was then selected for the following experiments. Based on the optimal mobile phase, a highly symmetrical and sharp characteristic peak of brimonidine tartrate was further obtained on Diamonsil C18 column (with better resolution, peak shapes and theoretical plates).

**Table 2:** The optimisation of the pH of phosphate buffer (buffer:methanol is 85:15)

Mobile phase	Theoretical plates (N)	Symmetry	Retention time (t_R_) (min)	Peak shape
pH 2.5	9485	0.85	5.020	+
pH 2.8	8119	0.90	4.273	+++
pH 3.0	8218	0.93	4.270	+++
pH 3.5	8360	0.95	4.276	+++
pH 4.0	8076	0.90	4.262	+++
pH 5.0	7774	0.90	4.242	+++
pH 6.0	7551	0.90	4.241	+++
pH 7.0	7338	0.91	4.268	+++
pH 8.0	7178	0.88	7.861	+

+, good peak shape

### Method validation

The developed chromatographic method was validated using ICH guidelines. Validation parameters included linearity, accuracy, precision, specificity, robustness, LOD and LOQ.

#### Linearity

Linearity was verified by a triplicate analysis of different concentrations of brimonidine tartrate solution. As a result, the linear regression equation was found to be Y = 103.42X + 2.83 (R^2^ = 0.9998, *n* = 7, 0.01–50 μg/mL) for brimonidine tartrate. In which, Y was the dependent variable, X was independent variable, 103.42 was slope which showed change in dependent (Y) variable per unit change in independent (X) variable; 2.83 was the Y-intercept i.e., the value of Y variable when X = 0. The linearity of developed chromatographic method was validated to be very good.

#### Accuracy

Accuracy of the developed method was determined by analyzing samples before and after adding some known amount of brimonidine tartrate. The acceptable recovery was set as between 97.0 and 103.0% and the results of accuracy confirmation of the proposed HPLC method were shown in Table 3.

**Table 3:** Accuracy of the proposed HPLC method

Spiked con. (μg/mL)	Measured con. (μg/mL) ± SD	Accuracy (%)	RSD (%)
0.1	0.098 ± 0.002	97.9	1.8
5	4.98 ± 0.02	99.7	0.4
50	49.96 ± 0.32	99.9	0.6

*SD* standard deviation, *RSD* relative standard deviation, *Con.* concentration

The developed analytical method had a good accuracy with overall recovery rates in the range of 97.9–99.9% for the analyte with RSDs below 1.8%, indicating that the proposed method was to be highly accurate and suitable for intended use.

#### Precision

The precision was evaluated by analyzing the standard solutions of brimonidine tartrate at three concentrations under the optimal conditions. It was considered at two levels: five times in one day for repeatability (intra-days) and on three consecutive days for intermediate precision (inter-days). The corresponding results were expressed as the relative standard deviation (RSD) and mean recovery of a series of measurements. The calculated RSD values of the intra-day and inter-day assays were <1.0 and 1.2%, respectively. The results of intra-day and inter-day precision of the proposed HPLC method were shown in Table 4. The results also demonstrated that brimonidine tartrate was stable in solution and the developed analytical method had high precision and was suitable for intended use.

**Table 4:** Intra-day and inter-day precision of the proposed HPLC method (*n* = 5)

Actual con. (μg/mL)	Intra-day precision	Inter-day precision
	Measured con. ± SD; RSD (%)	Measured con. ± SD; RSD (%)
0.1	0.098 ± 0.002; 1.5	0.099 ± 0.003; 1.5
5	4.98 ± 0.02; 0.4	4.98 ± 0.02; 2.1
50	49.96 ± 0.32; 0.6	49.98 ± 0.02; 1.3

*SD* standard deviation, *RSD* relative standard deviation, *Con.* concentration

#### Robustness

Robustness was validated by slightly varying the chromatographic conditions. The chromatographic conditions and corresponding results were shown in Tables 5. No obvious effects on the chromatographic parameters were observed in all of the deliberately varied chromatographic conditions (different flow rates, compositions of mobile phase and buffer pH).

**Table 5:** Robustness of the developed analytical method

Chromatographic condition	Assay %	t_R_ (min)	Theoretical plates	Symmetry
Flow rate (0.9 mL min^−1^)	98.0	4.747	8247	0.90
Flow rate (1 mL min^−1^)	99.7	4.279	7910	0.90
Flow rate (1.1 mL min^−1^)	102.7	3.868	7438	0.91
Methanol:buffer (12:88)	98.3	5.109	8218	0.93
Methanol:buffer (15:85)	99.7	4.270	7878	0.91
Methanol:buffer (18:82)	97.5	3.040	6898	0.90
Buffer (pH 3.0)	104.3	4.270	8218	0.93
Buffer (pH 3.5)	100.1	4.276	8360	0.95
Buffer (pH 4.0)	98.5	4.262	8076	0.90

#### LOD and LOQ

Based on a signal-to-noise ratio of 3:1, LOD was found to be 0.1 μg/mL for brimonidine tartrate. LOQ with a signal-to-noise of 10:1 was found to be 0.01 μg/mL for brimonidine tartrate.

#### Specificity

**Fig. 4:**
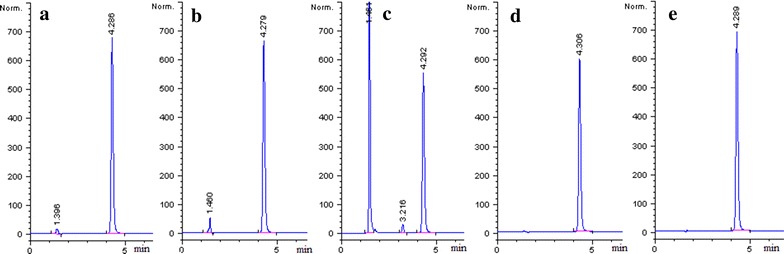
Chromatograms of brimonidine tartrate under **a** acidic stress, **b** basic stress, **c** oxidative stress, **d** thermal stress and **e** photolytic stress

Specificity was investigated by using photodiode array detection to ensure the homogeneity and evaluate peak purity which was evaluated at different stress conditions (acid, base, oxidation, thermal and photolytic) for brimonidine tartrate. The results were shown in Fig. 4.

Although several impurities and degradation products were detected, there was no influence on the main ingredients. The peak purity factor was more than 980 for drug product (Table 6), which further confirmed the specificity of this method.

**Table 6:** Stress testing results of brimonidine tartrate in stock solution

Nature of stress	Storage conditions	Time (h)	Amount [remaining ± SD (%)] (PP)	Extent of decomposition
5 M HCl	40 °C	24	96.5 ± 0.8 (999.74)	A little
5 M NaOH	40 °C	2	95.6 ± 2.3 (999.98)	A little
6% H_2_O_2_	40 °C	24	42.4 ± 0.5 (999.74)	Substantial
Thermal	40 °C	120	99.3 ± 0.2 (999.99)	None
	40 °C	240	99.2 ± 0.2 (999.99)	None
Dry heat	105 °C	7	99.4 ± 0.2 (999.85)	None
Photolytic	12 million lux hours and 200 W h/m^2^		99.3 ± 0.2 (999.99)	None

*n* = 3; PP = peak purity factor, peak purity factor value in the range of 980–1000 indicates a homogeneous peak

### Forced degradation study

All the stress conditions applied were enough to degrade brimonidine tartrate and other impurities in the pharmaceutical formulation. The results of stress studies are shown in Fig. 4 and Table 6. Brimonidine tartrate was degraded and remained ~96.5% when 5 M HCl was used at 40 °C for 24 h. Brimonidine tartrate remained ~95.6% when 5 M NaOH was used at 40 °C for 2 h. Brimonidine tartrate was degraded and only remained ~42.4% under 6% H_2_O_2_ at 40 °C for 24 h. The results of thermal stress showed that brimonidine tartrate was stable for 120 h under thermal stress (40 and 90 °C), even stable for 7 h under dry heat stress (105 °C). Brimonidine tartrate was not degraded substantial under photolytic stress. From these stress studies it was thus concluded that brimonidine tartrate was not stable in strong basic, strong acidic, especially oxidative conditions, but stable in thermal, dry heat and photolytic conditions. These results demonstrated that brimonidine tartrate could be used in the BRI/PHEMA drug delivery contact lens. The developed method effectively separated brimonidine tartrate from the impurities (Fig. 4). Therefore, the developed method can be considered highly specific for intended use.

### Application of the developed method

Application of the developed method was checked by analyzing brimonidine tartrate in commercially available pharmaceutical formulations and the BRI/PHEMA formulation. The results of commercial eye drops are provided in Table 7. The results showed high percentage recoveries and low RSD (%) values for commercial brimonidine tartrate eye drops.

**Table 7:** Assay results of brimonidine tartrate in commercial eye drops (*n* = 3)

Batch no.	Labled	Found	RSD (%)
E73767	10 mg 5 mL^−1^	9.74 mg 5 mL^−1^	1.41
E73506		9.66 mg 5 mL^−1^	1.15

The measured concentrations of brimonidine tartrate after a BRI/PHEMA drug delivery contact lens was leached for 1 h, 24 h and 7 day were 0.05, 0.04 and 0.01 μg/mL, respectively. It showed that brimonidine tartrate can be sustained released from the contact lens without a substantial fluctuating concentration. Thus, it can improve drug therapeutic efficacy. The results further confirmed that the developed method was suitable for drug analysis of brimonidine tartrate in the BRI/PHEMA drug delivery contact lenses and pharmaceutical formulations.

## Conclusion

A rapid and efficient RP-HPLC method was developed for the estimation of brimonidine tartrate in the BRI/PHEMA drug delivery contact lenses and pharmaceutical formulations. The proposed method was demonstrated to be linear, accurate, precise, robust and specific, based on method validation. Satisfactory results were obtained in separating the peaks of active pharmaceutical ingredients from the degradation products produced by forced degradation. Furthermore, the new method are cost-effective without the requirement of ion pairing and other derivatization agents, which are tend to adsorb very strongly on the stationary phase, resulting in difficulty in recovering initial column properties. Overall, the method is stability-indicating and can be used for routine analysis of brimonidine tartrate in quality control and any kind of stability and validation studies.

## Abbreviations

BRI: brimonidine tartrate
HEMA: 2-hydroxyethyl methacrylate
BRI/PHEMA: brimonidine/Poly(2-hydroxyethyl methacrylate)
IOP: intraocular pressure
PEG-DMA: poly (ethylene glycol) dimethacrylate
PDMS: polydimethylsiloxane
LOD: limit of detection
LOQ: limit of quantitation
ICH: International Conference on Harmonisation
HPLC: high performance liquid chromatography
